# Association of ATP-Binding Cassette Transporter A1 Gene Polymorphisms in Type 2 Diabetes Mellitus among Malaysians

**DOI:** 10.1155/2015/289846

**Published:** 2015-09-14

**Authors:** Polin Haghvirdizadeh, Vasudevan Ramachandran, Ali Etemad, Farzad Heidari, Nooshin Ghodsian, Norzian Bin Ismail, Patimah Ismail

**Affiliations:** ^1^Genetic Research Group, Department of Biomedical Science, Faculty of Medicine and Health Sciences, Universiti Putra Malaysia, 43400 Serdang, Selangor, Malaysia; ^2^Institute of Gerontology, Universiti Putra Malaysia, 43400 Serdang, Selangor, Malaysia; ^3^Department of Medicine, Faculty of Medicine and Health Sciences, Universiti Putra Malaysia, 43400 Serdang, Selangor, Malaysia

## Abstract

*Background*. Type 2 diabetes mellitus (T2DM) is a complex polygenic disorder characterized by impaired insulin resistance, insulin secretion, and dysregulation of lipid and protein metabolism with environmental and genetic factors. ATP-binding cassette transporter A1 (ABCA1) gene polymorphisms are reported as the one of the genetic risk factors for T2DM in various populations with conflicting results. This study was conducted based on PCR-HRM to determine the frequency of ABCA1 gene by rs2230806 (R219K), rs1800977 (C69T), and rs9282541 (R230C) polymorphisms Malaysian subjects. *Methods*. A total of 164 T2DM and 165 controls were recruited and their genotypes for ABCA1 gene polymorphisms were determined based on the real time high resolution melting analysis. *Results*. There was a significant difference between the subjects in terms of age, BMI, FPG, HbA1c, HDL, LDL, and TG (*P* < 0.05). There was a significant association between HOM of R219K (*P* = 0.005), among Malaysian subjects; moreover, allele frequency revealed the significant difference in A allele of R219K (*P* = 0.003). But, there was no significant difference in genotypic and allelic frequencies of C69T and R230C polymorphism. *Conclusion*. R219K polymorphism of ABCA1 gene can be considered as a genetic risk factor for T2DM subjects among Malaysians.

## 1. Background

Diabetes is one of the four main noncommunicable diseases (NCDs) identified by the World Health Organization (WHO), besides the cardiovascular disease, cancer, and chronic respiratory diseases [1]. Asia has become the major site of a quickly emerging diabetes epidemic. In Malaysia, the latest National Health and Morbidity Survey in 2008 already demonstrated an increase of prevalence of diabetes mellitus from 8.3% to 11.6% [2]. Type 2 diabetes mellitus (T2DM) is a complex polygenic disorder characterized by impaired insulin resistance, insulin secretion, and dysregulation of lipid and protein metabolism with environmental and genetic factors [3]. The complications of diabetes have become the leading causes of mortality and morbidity worldwide [4]. The prevalence of T2DM is swiftly increasing to 300 million people by the year 2025 [5]. Although several association studies suggested various candidate genes for T2DM in different populations, the role of genetic polymorphisms involved in the development of diabetes still remains unclear [6]. The ATP-binding cassette transporter A1 (ABCA1) gene located on the chromosome 9 region q31.1 involved in the transport of cholesterol and phospholipids from cells to lipid-poor apolipoproteins [7]. Besides, some investigations demonstrated that familial hypoalphalipoproteinemia can be caused by heterozygosity and Tangier disease can be caused by homozygous mutations. Moreover, genetic and molecular biology researchers have suggested that low plasma HDL-cholesterol can reflect an impaired ABCA1 pathway that can also elevate the accumulation of cholesterol in tissue macrophages [8].

Analyzing the genetic and allelic frequency of polymorphisms among case and control studies can provide supporting evidence to show their role in individual subjects. The genetic polymorphisms of ABCA1 gene provide a basis for studying the association between genetic variants and the development of T2DM in this ethnic group. Based on literature review, most of the studies were done on 3 common polymorphisms, so this study was conducted to determine genetic polymorphism of the rs2230806 (R219K), C69T (rs1800977), and R230C (rs9282541) among T2DM subjects.

## 2. Results 

A total of 329 subjects were approached in this study that were divided into 2 groups: 165 controls and 164 T2DM subjects. In this study, the association of anthropometric and social demographic factors and major risk factors in T2DM with ABCA1 polymorphism among Malaysian subjects was investigated.

The means of age, BMI, systolic blood pressure (SBP), FPG, HbA1c, HDL, and triglyceride (TG) among T2DM patients were higher than control group; moreover, diastolic blood pressure (DBP), low density lipoprotein (LDL), and cholesterol in control subjects were higher than T2DM patients. The control samples had mean age 54.97 ± 11.79 and patients had mean age 62.14 ± 9.56. Mean BMI were almost equal: 27.08 ± 6.26 in control and 28.60 ± 13.98 in case samples. Mean of SBP among controls was 137.89 ± 20.22 and among case subjects was 141.83 ± 24.41 and mean of DBP was 79.54 ± 10.52 between control groups and 78.32 ± 12.14 between case subjects. Mean of FPG was higher among case subjects (8.66 ± 3.50) and in control samples was 5.45 ± 1.31. Mean of HbA1c among patients (7.90 ± 2.08) is higher than controls (5.94 ± 0.65). The means of cholesterol, LDL, HDL, and TG between case and control subjects are almost equal. Maximum age between case and control subjects is 85 years and minimum age in control is 26 years and in case is 41 years. Based on this table the significant difference was detected in the level of age (*P* value = 0.000), FPG (*P* value = 0.000), HbA1c (*P* value = 0.000), LDL (*P* value = 0.010), and TG (*P* value = 0.000) between T2DM and control subjects (Table 1).

### 2.1. R219K

**Figure 1:**
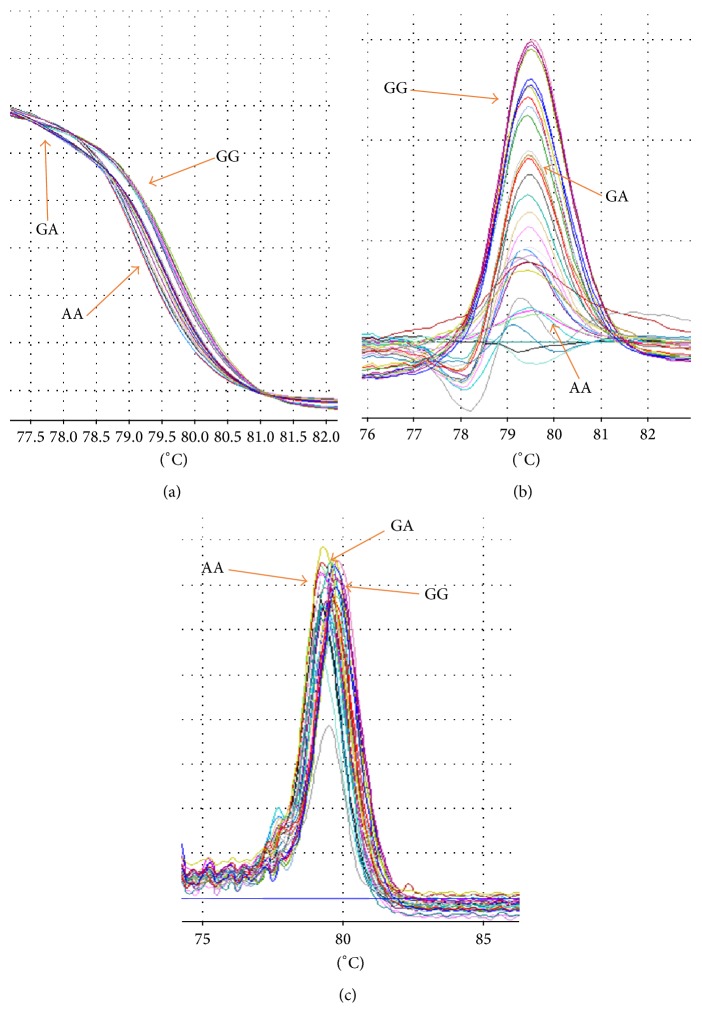
The difference graph of HRM analysis for the genotypes of R219K polymorphism. (a) demonstrated the normalized graph, (b) revealed the difference graph, and (c) was melting graph. In these graphs WT had higher Tm and HOM had lower Tm.

Analysis of R219K polymorphism with 73 bp PCR product with mutation (G→A) was amplified by PCR-HRM.  Figure 1 showed three different graphs that can detect three different genotypes of R219K polymorphism. The genotypic and allelic frequencies of R219K were shown in Table 2 that demonstrated the significant difference genotypes of this polymorphism. The clinical and biochemical characteristics were demonstrated in Table 3 for this polymorphism. Based on this table the *P* value showed FPG (0.036) was significantly higher in WT in both groups. There was significantly higher difference in the mean of HbA1c of WT for both subjects (*P* = 0.001).

### 2.2. C69T

**Figure 2:**
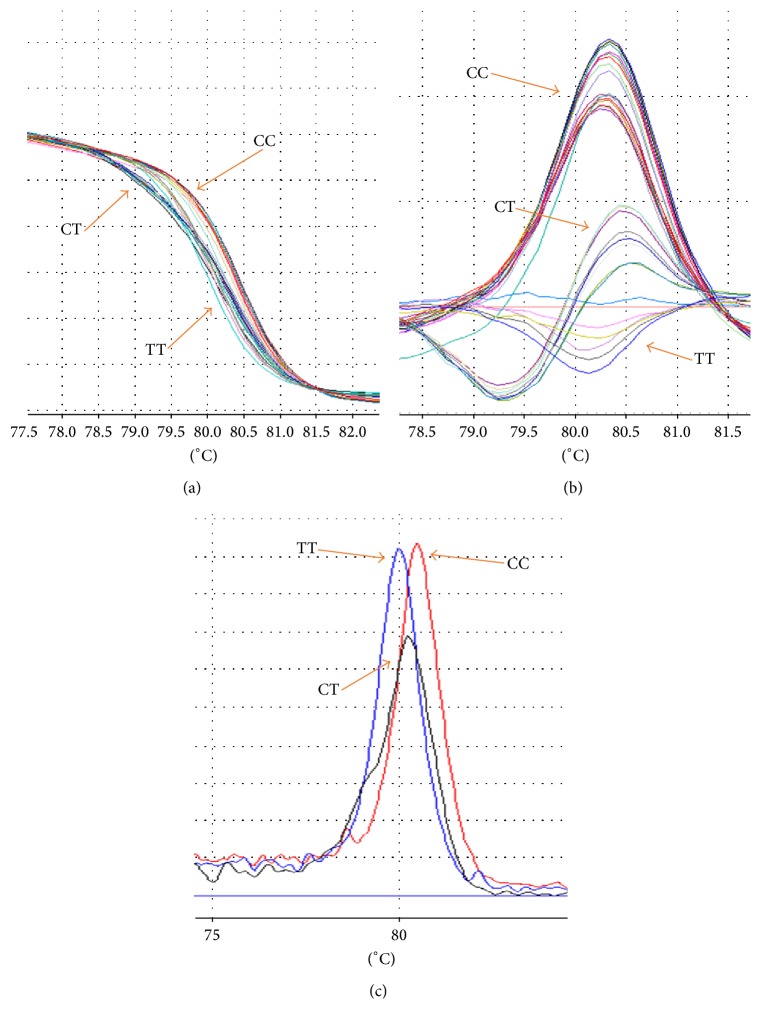
The difference graph of HRM analysis for the genotypes of C69T polymorphism. (a) demonstrated the normalized graph, (b) revealed the difference graph, and (c) was melting graph. In these graphs WT had higher Tm and HOM had lower Tm.

Analysis of C69T polymorphism with 85 bp PCR product length with mutation (C→T) was amplified by PCR-HRM.  Figure 2 showed three different graphs that can detect three different genotypes of C69T polymorphism. The genotypic and allelic frequencies of C69T were shown in Table 2 that did not demonstrate any differences for this polymorphism. The clinical and biochemical characteristics of this polymorphism were demonstrated in Table 4.

### 2.3. R230C

**Figure 3:**
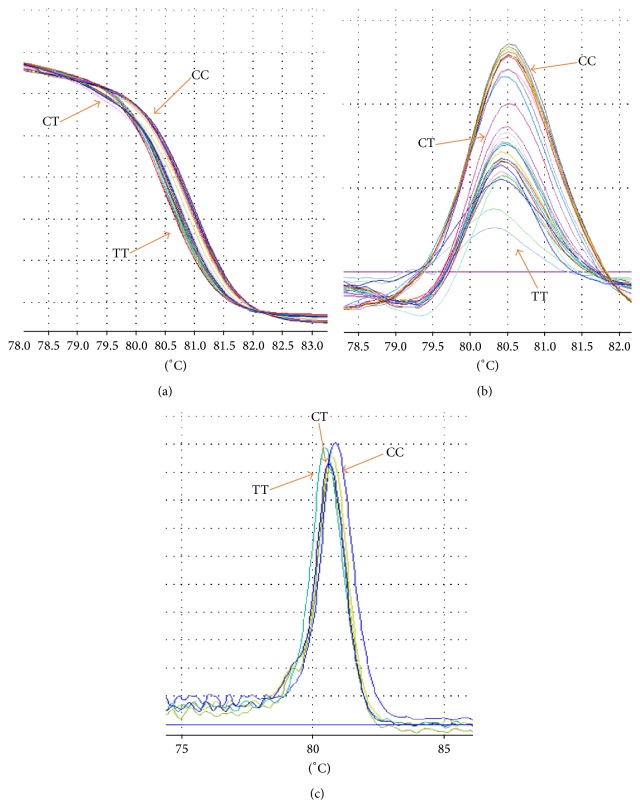
The difference graph of HRM analysis for the genotypes of R230C polymorphism. (a) demonstrated the normalized graph, (b) revealed the difference graph, and (c) was melting graph. In these graphs WT had higher Tm and HOM had lower Tm.

Analysis of R230C polymorphism with 87 bp PCR product length with mutation (C→T) was amplified by PCR-HRM.  Figure 3 showed three different graphs that can detect three different genotypes of R230C polymorphism. The genotypic and allelic frequencies of R230C were shown in Table 2 that did not demonstrate any differences for this polymorphism. The clinical and biochemical characteristics of this polymorphism were demonstrated in Table 5.

## 3. Discussion

As revealed in some investigations, the worldwide prevalence of diabetes has gone up and this represents the importance of the study on diabetes; moreover, in Malaysia, the latest National Health and Morbidity Survey in 2008 has already demonstrated an increase in the prevalence of diabetes mellitus from 8.3% to 11.6% [19]. The proportion of Malays was higher in both T2DM and control subjects in this study (50.6% and 50.3%, resp.) in comparison with Indians and Chinese (29.9% and 30.3%, resp., for Indians and 19.5% and 19.4%, resp., for Chinese). Moreover, the proportion of males was higher in T2DM and control subjects (62.8% and 52.7%, resp.) in comparison with females (37.2% and 47.3%, resp.). The significant difference in the levels of age (*P* = 0.000), FPG (*P* = 0.000), LDL (*P* = 0.010), and TG (*P* = 0.000) was observed among the subjects. In previous reports a significant difference was detected in the levels of BMI, HDL, LDL, TG, cholesterol, FPG, SBP, and DBP between T2DM and healthy subjects [8, 13]. Among overweight/obese population demographic and clinical characteristics were evaluated which demonstrated no significant differences in the level of HDL and LDL but statistical differences were detected in the levels of BMI, cholesterol, and TG [16]. In the current study, there were no significant differences between genotypes and subjects for R230C and C69T polymorphisms. But, regarding R219K there was a significant difference between subjects. Allele frequencies of these polymorphisms revealed no significant difference of R230C but there was a significant difference between alleles of R219K and C69T. Based on genotype and allele frequency of polymorphisms according to gender among subjects, there was no significant difference between genotypes and gender among subjects for any of the polymorphisms but A allele of R219K and C allele of C69T and T allele of R230C had significant differences between genders among subjects.

Genotype and allele frequencies of each polymorphism revealed no significant difference for genotype and allele of C69T and R230C according to race among subjects but R219K had a significant difference in HOM and A allele. R219K was significantly different between metabolic syndrome patients and healthy subjects based on Değer's group research [20].

Opposite to those studies, some researchers showed the significant differences between T2DM and controls: they found TT genotype was significantly higher in controls than in patients, but Alharbi and his colleagues showed the frequency of T allele was higher among patients and Ergen et al. found that T allele was higher in control groups; moreover these 2 groups did not find any association between C69T and lipid profiles [8, 13]. R230C polymorphism of ABCA1 gene associated with lower HDL; moreover, C230 allele had a significant association with lower cholesterol and HDL level as well. However, no association was detected between C230 allele and T2DM [15].

Among overweight/obese and control populations there was no significant difference for genotype frequency [16].  Table 6 demonstrated the conflicting results of genetic variants of ABCA1 gene found in different populations compared with the current study.

## 4. Conclusion

The present study has showed a genetic association for R219K, C69T, and R230C polymorphisms of ABCA1 gene among Malaysian T2DM patients compared to control subjects. Replication studies with a larger number of samples on a homogenous study population are strongly recommended to confirm the association of ABCA1 gene polymorphisms with T2DM.

In the current study, some limitations have been considered. The present study provided only genetic association of ABCA1 gene polymorphisms with T2DM among Malaysian subjects. Apart from the ABCA1 gene polymorphisms, other polymorphisms such as A2589G, G3456C, R1851X, and K776N need to be analyzed to determine the association of the other candidate gene polymorphisms with T2DM and other defects.  Table 6 shows the conflicting results of genetic variants found in different populations.

## 5. Methods

### 5.1. Study Subjects

Ethical approval has been obtained from the Ethical Committee of the National Heart Institute, Malaysia (Reference number IJNEC/05/10 (02)). A total of 164 T2DM subjects were collected from the National Heart Institute, Kuala Lumpur. Subjects were selected as adults > 30 years of age who were already diagnosed with T2DM and participated in IJN for their T2DM treatment. A questionnaire in both Malay and English languages was obtained to assess the sociodemographic factors. Informed consent was obtained from all the subjects who have participated in this study. A total of 165 control individuals were recruited from the healthy respondents who did not have T2DM at the time of sample collection. The subjects who had been diagnosed with cancer, type 1 diabetes, genetic malformation, and pregnancy are excluded from this study.

### 5.2. Genomic DNA Extraction

The buccal and blood samples were collected from the respective subjects. The genomic DNA was extracted from blood and buccal samples by using the extraction kits (QIGEN) and used for amplification of the candidate genes and then the extracted genomic DNA was stored at −20°C for further analysis. PCR optimization was done first for all primers, followed by DNA qualifications. The concentration of the extracted DNA was estimated using the NanoDrop in two OD wavelengths 260 nm and 280 nm.

### 5.3. Genotyping of ABCA1 Gene Polymorphisms

Genotyping of these polymorphisms was done based on real time PCR-HRM and the primers were designed and synthesized by NextGene. The PCR product of samples that were amplified from the respective genes was sequenced and confirmed the product. Those samples were used as a positive control for the respective genes. The negative control consisted of PCR-grade water lacking the DNA template. All samples were genotyped by high resolution melting analysis by Rotor Gene 6000. PCR primers for R219K were forward primer CCATGTTGGAACGAAGTA and reverse primer GAAGTTTCTGAGCTTTGTG, for C69T were forward primer TCTCGCTCGCAATTACGG and reverse primer TGACCGATAGTAACCTCTGC, and for R230C were forward primer GATTGGCTTCAGGATGTCCATGTTG and reverse primer GTTTCTGAGCTTTGTGGCCT ACC. PCR was performed in a volume of 25 *μ*L consisting of about 1 *μ*L of genomic DNA, 0.5 *μ*L of each of the forward and reverse primers, 13 *μ*L of water, and 10 *μ*L Master Mix for HRM (EpiTect HRM Master Mix, QIGEN). The temperature was kept at 95 for 5 min for denaturation followed by 40 cycles of 20 s at 95°C, 10 s at 55°C, and 20 s at 72°C. The melting curves were normalized (temperature ranges on each side of the melting transition were chosen and the data points for a given sample were scaled between 0 and 100% fluorescent intensity). Used control samples (wild type (WT) and homozygous mutant (HOM)) were confirmed by sequencing. Table 1 demonstrated the sequence and length and Tm of each primer.

### 5.4. Statistical Analysis

The Statistical Package for Social Science (SPSS) (Version 21) was used to analyze the data in this study. Descriptive statistics were utilized to analyze all variable information such as demographics, anthropometric factors, and the genotypes of all the study subjects; moreover, all of these factors were compared by using Student's *t*-test; one-way ANOVA test was utilized to compare the group means and a level of *P* < 0.05 was considered as statistically significant. Confidence intervals (95%) were reported where appropriate. Allelic frequencies were calculated by the gene counting method and the genotype distribution was calculated with Hardy-Weinberg expectations by a chi-squared test.

## Figures and Tables

**Table 1:** Clinical and biochemical characteristics between T2DM and control subjects.

Parameters	T2DM (*n* = 164)	Controls (*n* = 165)	*P* value
Age (years)	62.14 ± 9.56	54.97 ± 11.79	0.000^*^
BMI (Kg/m²)	27.86 ± 5.14	27.08 ± 6.26	0.22
WHR	0.96 ± 0.15	0.95 ± 0.56	0.876
SBP (mm Hg)	141.83 ± 24.41	137.89 ± 20.22	0.117
DBP (mm Hg)	78.30 ± 12.14	79.55 ± 10.52	0.325
FPG (mmol/L)	8.66 ± 3.50	5.45 ± 1.31	0.000^*^
HbA1c (mmol/mol)	7.98 ± 2.18	5.94 ± 0.65	0.000^*^
LDL (mmol/L)	2.35 ± 0.90	2.62 ± 0.95	0.010^*^
HDL (mmol/L)	1.26 ± 0.48	1.21 ± 0.43	0.303
TG (mmol/L)	1.76 ± 1.20	1.27 ± 0.76	0.000^*^
Cholesterol (mmol/L)	4.38 ± 1.14	4.43 ± 1.17	0.675

Values shown are the mean ± SD, *P* < 0.05.

^*^Significant parameters.

**Table 2:** Genotypic and allelic frequencies of R219K, C69T, and R230C, respectively, according to subjects.

		T2DM (%)	(95% CI)	Controls (%)	*P* value
R219K	GG	94 (57.3%)		56 (33.9%)	
Genotypes and alleles	GA	55 (33.5%)		77 (46.6%)	0.000^*^
	AA	15 (9.1%)		32 (19.3%)	
Alleles	G	243 (74%)		189 (57.2%)	0.000^*^
	A	85 (26%)		141 (42.8%)	

	GG versus GA		0.07–0.34		0.001^*^
Post hoc test	GG versus AA		0.11–0.49		0.001^*^
	GA versus AA		−0.09–0.29		0.467

C69T	CC	65 (39.6%)		83 (50.3%)	
Genotypes and alleles	CT	48 (29.2%)		48 (29%)	NS
	TT	51 (31%)		34 (20.6%)	
	C	154 (54.2%)		190 (64.8%)	0.005^*^
Alleles	T	150 (45.8%)		116 (35.2%)	

	CC versus CT		−0.18–0.06		0.352
Post hoc test	CC versus TT		−0.29–−0.02		0.018^*^
	CT versus TT		−0.24–0.04		0.179

R230C	CC	69 (42%)		64 (38.7%)	
Genotypes and alleles	CT	72 (43.6%)		67 (40.6%)	NS
	TT	32 (19.3%)		34 (20.6%)	
	C	210 (63.9%)		195 (59%)	0.695
Alleles	T	136 (36.1%)		135 (41%)	

	CC versus CT		0.11-0.12		0.989
Post hoc test	CC versus TT		−0.04–0.27		0.147
	CT versus TT		−0.04–0.26		0.147

^*^ *P* < 0.05.

**Table 3:** Clinical and biochemical characteristics among subjects according to genotypes of R219K.

	T2DM (*n* = 164)			Controls (*n* = 165)			*P* value
	GG	GA	AA	GG	GA	AA	
Age (years)	61.94 ± 9.20	61.40 ± 9.98	66.06 ± 9.76	54.94 ± 11.79	55.38 ± 12.11	54.06 ± 10.98	0.583
BMI (Kg/m²)	27.68 ± 5.12	28.32 ± 5.54	27.29 ± 3.69	27.53 ± 8.86	27.17 ± 4.44	26.08 ± 4.24	0.767
WHR	0.95 ± 0.07	0.97 ± 0.25	0.96 ± 0.06	0.88 ± 0.13	1.02 ± 0.82	0.91 ± 0.06	0.791
SBP (mm Hg)	145.45 ± 22.85	134.98 ± 25.86	145.85 ± 24.12	136.72 ± 20.59	138.12 ± 19.44	139.34 ± 21.93	0.479
DBP (mm Hg)	78.21 ± 10.14	78.44 ± 15.15	78.28 ± 11.41	79 ± 11.21	79.36 ± 10.12	80.96 ± 10.43	0.481
FPG (mmol/L)	8.76 ± 3.66	8.65 ± 3.39	8.11 ± 3.04	5.62 ± 1.14	5.35 ± 1.24	5.42 ± 1.69	0.036^*^
HbA1c (mmol/mol)	8.22 ± 2.14	7.76 ± 2.15	7.24 ± 2.46	5.92 ± 0.69	5.91 ± 0.63	6.07 ± 0.65	0.001^*^
LDL (mmol/L)	2.34 ± 0.92	2.33 ± 0.87	2.46 ± 0.94	2.71 ± 0.95	2.47 ± 0.91	2.80 ± 1.03	0.138
HDL (mmol/L)	1.24 ± 0.40	1.27 ± 0.54	1.31 ± 0.69	1.28 ± 0.44	1.19 ± 0.46	1.10 ± 0.29	0.382
TG (mmol/L)	1.85 ± 1.45	1.58 ± 0.76	1.90 ± 0.82	1.26 ± 0.79	1.29 ± 0.81	1.25 ± 0.58	0.139
Cholesterol (mmol/L)	4.42 ± 1.19	4.29 ± 1.08	4.45 ± 1.11	4.66 ± 1.16	4.30 ± 1.21	4.34 ± 1.04	0.218

^*^ *P* < 0.05.

**Table 4:** Clinical and biochemical characteristics among subjects according to genotypes of C69T.

	T2DM (*n* = 164)			Controls (*n* = 165)			*P* value
	CC	CT	TT	CC	CT	TT	
Age (years)	61.93 ± 9.65	62.55 ± 10.10	62.04 ± 9.09	55.60 ± 11.50	54.51 ± 12.62	54.06 ± 11.55	0.958
BMI (Kg/m²)	27.99 ± 5.07	27.98 ± 5.53	27.58 ± 4.94	26.98 ± 7.32	27.05 ± 5.22	27.35 ± 4.72	0.992
WHR	0.97 ± 0.23	0.95 ± 0.65	0.95 ± 0.07	1.00 ± 0.87	0.88 ± 0.05	0.91 ± 0.07	0.544
SBP (mm Hg)	142.79 ± 20.84	143.76 ± 25.25	138.94 ± 27.58	137.26 ± 19.17	137.10 ± 20.16	140.63 ± 23.11	0.961
DBP (mm Hg)	79.20 ± 14.04	79.69 ± 11.35	75.96 ± 10.16	79.13 ± 10.06	80.43 ± 11.34	79.33 ± 10.64	0.256
FPG (mmol/L)	8.81 ± 3.27	8.49 ± 3.64	8.64 ± 3.72	5.63 ± 1.57	5.21 ± 1.03	5.35 ± 0.79	0.592
HbA1c (mmol/mol)	7.95 ± 2.29	8.11 ± 1.93	7.88 ± 2.32	5.92 ± 0.68	5.95 ± 0.55	5.96 ± 0.72	0.575
LDL (mmol/L)	2.32 ± 0.91	2.47 ± 0.97	2.26 ± 0.81	2.57 ± 1.00	2.54 ± 0.93	2.85 ± 0.86	0.916
HDL (mmol/L)	1.22 ± 0.49	1.32 ± 0.46	1.26 ± 0.50	1.28 ± 0.43	1.10 ± 0.39	1.18 ± 0.45	0.788
TG (mmol/L)	1.93 ± 1.60	1.56 ± 0.63	1.73 ± 0.99	1.23 ± 0.86	1.21 ± 0.62	1.48 ± 0.67	0.291
Cholesterol (mmol/L)	4.39 ± 1.26	4.44 ± 1.08	4.31 ± 1.05	4.41 ± 1.18	4.29 ± 1.18	4.69 ± 1.11	0.843

*P* value < 0.05, between T2DM and controls.

**Table 5:** Clinical and biochemical characteristics among subjects according to genotypes of R230C.

	T2DM (*n* = 164)			Controls (*n* = 165)			*P* value
	CC	CT	TT	CC	CT	TT	
Age (years)	62.19 ± 8.82	62.04 ± 10.03	62.30 ± 10.55	56.68 ± 10.60	52.80 ± 12.58	56.00 ± 12.00	0.363
BMI (Kg/m²)	28.01 ± 5.33	27.66 ± 5.27	28.00 ± 4.26	28.60 ± 8.05	26.10 ± 4.53	26.14 ± 4.72	0.096
WHR	0.94 ± 0.05	0.98 ± 0.22	0.93 ± 0.05	1.02 ± 0.94	0.92 ± 0.06	0.91 ± 0.08	0.730
SBP (mm Hg)	143.53 ± 25.25	140.19 ± 24.75	142.25 ± 20.98	139.28 ± 20.19	135.04 ± 18.38	140.82 ± 23.40	0.351
DBP (mm Hg)	78.53 ± 13.96	77.87 ± 11.33	79.10 ± 8.65	79.70 ± 10.50	78.83 ± 10.46	80.67 ± 10.87	0.615
FPG (mmol/L)	8.66 ± 3.71	8.88 ± 3.32	7.93 ± 3.49	5.42 ± 0.92	5.50 ± 1.74	5.42 ± 0.90	0.193
HbA1c (mmol/mol)	8.10 ± 2.09	8.02 ± 2.22	7.41 ± 2.38	5.83 ± 0.67	6.01 ± 0.69	6.00 ± 0.52	0.192
LDL (mmol/L)	2.58 ± 1.01	2.18 ± 0.78	2.13 ± 0.73	2.59 ± 0.98	2.72 ± 0.97	2.48 ± 0.87	0.241
HDL (mmol/L)	1.33 ± 0.51	1.23 ± 0.47	1.12 ± 0.36	1.18 ± 0.41	1.23 ± 0.34	1.20 ± 0.60	0.494
TG (mmol/L)	1.78 ± 1.08	1.70 ± 1.23	1.89 ± 1.42	1.29 ± 0.78	1.32 ± 0.83	1.14 ± 0.54	0.968
Cholesterol (mmol/L)	4.69 ± 1.22	4.16 ± 1.07	4.08 ± 0.88	4.40 ± 1.24	4.56 ± 1.09	4.25 ± 1.17	0.119

*P* value < 0.05, between T2DM and controls.

**Table 6:** Conflicting results of genetic variants found in different populations.

Gene variants	Diseases	Populations	References	Number of subjects	*P* value
ABCA1 R1587K	CAD	Chinese	Guo et al., 2011 [9]	222	NS
ABCA1 R219K	CHD	Turkish	Çoban et al., 2014 [10]	627	NS
ABCA1 C69T	CAD	Turkish	Ergen et al., 2008 [11]	127	NS
ABCA1 R219K	CHD	Spanish	Cenarro et al., 2003 [12]	216	S
ABCA1 C69T	T2DM	Saudi Arabian	Alharbi et al., 2013 [13]	756	S
ABCA1 C69T	T2DM	Turkish	Ergen et al., 2012 [8]	157	S
ABCA1 R219K, R1587K, C69T	T2DM	French	Porchay-Baldérelli et al., 2009 [14]	482	NS
ABCA1 R230C	CAD	Mexican	Villarreal-Molina et al., 2012 [15]	2193	S
ABCA1 R219K	Overweight/obese	Thai	Kitjaroentham et al., 2007 [16]	229	NS
ABCA1 R219K, R1587K, C69T	Ischemic stroke	English	Pasdar et al., 2007 [17]	887	S
ABCA1 R1219K	CAD	American	Benton et al., 2007 [18]	6814	NS
R219K	T2DM	Malaysian	Current study	164	S

NS: nonsignificant (*P* > 0.05), S: significant (*P* < 0.05).

## Abbreviations

T2DM: Type 2 diabetes mellitus
ABCA1: ATP-binding cassette transporter A1
WT: Wild type
HET: Heterozygous
HOM: Homozygous
CI: Confidence interval.
